# Morphological Aspects of the Aberrant Right Subclavian Artery—A Systematic Review of the Literature

**DOI:** 10.3390/jpm14040335

**Published:** 2024-03-22

**Authors:** Alin Horatiu Nedelcu, Ancuta Lupu, Marius Constantin Moraru, Cristina Claudia Tarniceriu, Cristinel Ionel Stan, Simona Alice Partene Vicoleanu, Ana Maria Haliciu, Gabriel Statescu, Manuela Ursaru, Ciprian Danielescu, Ileana Ioniuc, Razvan Tudor Tepordei, Vasile Valeriu Lupu

**Affiliations:** 1 Department of Morpho-Functional Science I, Discipline of Anatomy, “Grigore T. Popa” University of Medicine and Pharmacy, 16 Universitatii Street, 700115 Iasi, Romania; alin.nedelcu@umfiasi.ro (A.H.N.); claudia.tarniceriu@umfiasi.ro (C.C.T.); cristinel.stan@umfiasi.ro (C.I.S.); partene.vicoleanu@umfiasi.ro (S.A.P.V.); ana.haliciu@umfiasi.ro (A.M.H.); gabriel.statescu@umfiasi.ro (G.S.); razvan.tepordei@umfiasi.ro (R.T.T.); 2 Radiology Clinic, Recovery Hospital, 700661 Iasi, Romania; 3 Department of Mother and Child, “Grigore T. Popa” University of Medicine and Pharmacy, 16 Universitatii Street, 700115 Iasi, Romania; ileana.ioniuc@umfiasi.ro (I.I.); vasile.lupu@umfiasi.ro (V.V.L.); 4 Department of Surgical Sciences I, “Grigore T. Popa” University of Medicine and Pharmacy, 16 Universitatii Street, 700115 Iasi, Romania; manuela.ursaru@umfiasi.ro (M.U.); ciprian.danielescu@umfiasi.ro (C.D.); 5 Radiology Clinic, “Sf Spiridon” County Clinical Emergency Hospital Iasi, 700661 Iasi, Romania

**Keywords:** lusoria artery, subclavian artery, dysphagia lusoria, aortic arch, vertebral artery, Kommerell diverticulum, bicarotid trunk

## Abstract

Background: The aberrant origin of the right subclavian artery (ARSA), also known as the lusoria artery, is a congenital malformation with an incidence of 0.5–4.4%. Most cases are incidental due to minimal clinical manifestations. Computer tomography (CT) is important in diagnosing and evaluating these patients. Materials and Methods: We conduct a computerized search in two databases, PubMed and EMBASE, for articles published between 1 January 2022 and 31 December 2023, PROSPERO code: CRD42024511791. Eligible for inclusion were case reports and case series that presented the aberrant origin of the right subclavian artery. The main outcome was the highlighting of the morphological types of ARSA. In this context, we proposed a new classification system of this anomaly. The secondary outcome was the evaluation of the demographic distribution of the lusoria artery. Results: Our search identified 47 articles describing 51 patients with ARSA. The typical course for ARSA is retroesophageal, being registered in 49 out of 51 patients. This malformation is frequently associated with Kommerell diverticulum (15 out of 51), troncus bicaroticus (7 out of 51), and aberrant origins of the right vertebral artery (7 out of 51). We observed a higher incidence of the condition among women (32 out of 51) compared to men (19 out of 51). From a demographic point of view, ARSA is more frequent in the “44 to 57 years” and “58 to 71 years” age ranges. Conclusions: ARSA is a congenital malformation resulting from a defect in the development of the aortic arches. The imaging studies such as computer tomography play a defined diagnostic role.

## 1. Introduction

Lusoria artery (LA) is a malformation of the right subclavian artery, which emerges directly from the aortic arch and not from the brachiocephalic trunk. David Bayford (1794) discovered this anomaly in a 62-year-old cadaver who suffered from prolonged dysphagia. He named the symptom “dysphagia lusoria”, which means “dysphagia by a freak of nature”. In fact, it is an extrinsic compression of the esophagus caused by a vascular anomaly of the aortic arch, mainly from an aberrant right subclavian artery (ARSA) [1]. For many years, the condition was also known as Bayford syndrome [2]. In 1936, Kommerell described on a radiograph the association between this malformation and the aneurysmal dilatation of the aortic arch known as the “Kommerell diverticulum” [3].

Along with the anomalous origin of the pulmonary arteries [4] and the anomalous pulmonary venous return [5], the aberrant right subclavian artery represents a congenital vascular anomaly of the great vessels of the heart. While the anomalous origin of the pulmonary artery has a prevalence of 0.1–0.33% [4] and the anomalous venous return of 0.8/10.000 of newborns [5], ARSA is rated with an incidence between 0.5% and 4.4% of live births [3,6,7].

The majority of cases (60–80%) is asymptomatic and it is an incidental discovery in imaging studies or during autopsy. More than half of the patients are symptom-free lifelong [3,6].

Our systematic review of the literature follows the morphological aspect of ARSA and its association with other vascular malformations, such as the origin of the right vertebral artery, the presence of the Kommerell diverticulum, and the bicarotic trunk. In addition, considering the morphological variants, we propose an original classification of ARSA. Secondly, we conducted an extensive review regarding the development of aortic arches.

## 2. Materials and Methods

### 2.1. Literature Search Strategy

**Figure 1:**
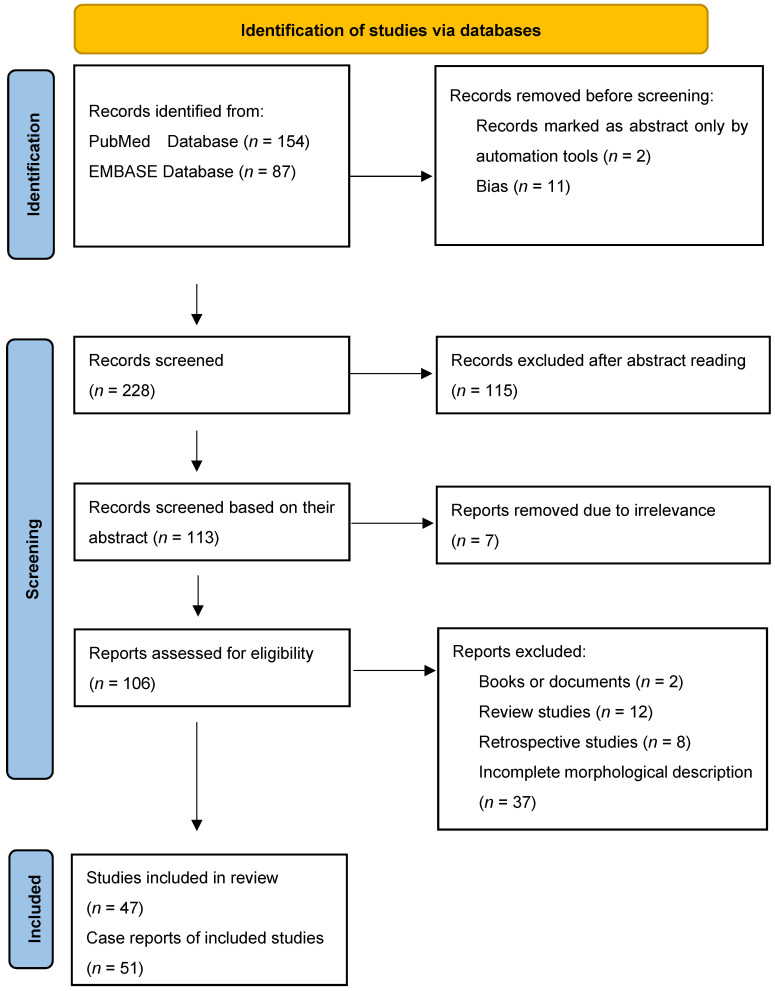
Literature search strategy.

The protocol for this systematic review was registered in the international register of systematic reviews—PROSPERO code: CRD42024511791. Two independent authors conducted the literature search in two databases: PubMed and EMBASE. We initiated the search protocol on 2 January 2024 for articles published between January 2022 and December 2023. The Pubmed search used the following keywords: “artera lusoria” OR “aberrant right subclavian artery”. The search query for EMBASE was set as follows: ‘artera lusoria’ OR ‘aberrant subclavian artery’. After reading the abstracts, we selected 78 articles from PubMed (out of 154) and 35 articles from EMBASE (out of 87). We applied the inclusion and exclusion criteria (Table 1), and 47 articles remained in this review. A third investigator who had the decisive vote resolved the differences of opinion between the investigators regarding the selection carried out. The search strategy is presented in a flow chart (Figure 1) following PRISMA guidelines [8].

### 2.2. Types of Studies

We selected human studies. Cadaveric studies were also accepted.

### 2.3. Types of Participants

We selected patients with aberrant right subclavian artery.

### 2.4. Types of Outcomes and Data Analyses

Each clinical case included in this review was studied from a morphological and clinical point of view. We traced the abnormal origin of the right subclavian artery, the type of trajectory, and the association with the Kommerell diverticulum or the bicarotid trunk. Another point of interest is the origin of the right vertebral artery due to great variability.

### 2.5. Data Extraction

We accessed the selected articles using “Gr. T. Popa” University of Medicine and Pharmacy VPN. We kept only the published full-text manuscripts in the study.

## 3. Results

This systematic review analyzed 47 articles describing the morphological features of the aberrant right subclavian artery (ARSA) and associated vascular malformations in a total of 51 patients aged between 1 and 84 years. In the group obtained, women predominated, compared to men (32 vs. 19). From a morphological point of view, in most cases, the path of the right subclavicular artery is retroesophageal (49 out of 51) and only two cases showed a pretracheal path. The association of the aberrant right subclavicular artery with the Kommerell diverticulum and the bicarotid trunk is frequent, with our study registering no less than 15 out of 51 patients and 7 out of 51 patients, respectively. Another point of interest of our study is represented by the origin of the right vertebral artery. In most of the included cases, RVA originates from ARSA (44 out of 51), being considered the normal origin. Seven cases have an aberrant origin, two in the aortic arch and five in the right common carotid artery, respectively. The symptoms are dominated by dysphagia (24 out of 51 patients), followed by chest pain (5 out of 51), weight loss (4 out of 51), and dyspnea (2 out of 51). No less than 7 patients were diagnosed accidentally, without presenting any symptoms. The outcomes of the studies are presented in Table 2.

### Data Analyses

The study group includes 51 patients, varying between the 1–84 years old range; the mean age is 64.56 years ± 35.07, and the median is 58.25 years. The distribution of frequencies by age groups is presented in Table 3.

The breakdown by gender showed a higher incidence of the condition among women (32 out of 51) compared to men (19 out of 51). No statistical significance was recorded (*p* = 0.1923).

Even if the symptomatology associated with this malformation is not specific, we have grouped the results into two categories: specific and non-specific. We have included, in the first category, the symptoms that are directly related to the mass effect exerted by the abnormal trajectory of ARSA, such as dysphagia, dyspnea, chest pain, and foreign body sensation (Table 4).

We classified all other symptoms, including weight loss, and asymptomatic patients in the non-specific category. The resulting data are presented in tabular form (Table 5).

## 4. Discussion

The development of the blood vessels is correlated with two main processes: vasculogenesis and angiogenesis. Often, the two terms are used interchangeably. However, they describe different developmental processes. Vasculogenesis represents new blood vessel formation. It starts on the eighteenth day of embryonic development when specific cells of the intraembryonic mesoderm differentiate into angioblasts. They agglutinate to form small vesicles. Furthermore, the vesicles fuse into tubes and establish the circulatory system of the embryo [55]. The second process, angiogenesis, consists of budding and sprouting at the very end of the initial vessels of the embryo. From this point on, the two processes coexist with the splitting or fusion of the new blood vessels, or even forming anastomoses such as those at the cerebral level. These processes can be the basis for the occurrence of a vascular malformation such as ectopic origin, duplication, or fenestration [55,56].

### 4.1. Embryological Development of Aortic Arches—Starting Point in the Elucidation of Great Vessel Malformations

Vasculogenesis plays a prominent role during heart development because it is the primary process involved in forming lateral endocardial tubes. Lately, they will fuse to form the primary cardiac tube (PCT). The cranial segment of the primary cardiac tube will form the two ventral aortas that loop dorsally [55,57,58].

The paired dorsal aortas right (RDA) and left (LDA), are born through the same vasculogenesis process. The angioblasts develop in the dorsal mesenchyme of the paraxial mesoderm on either side of the notochord. The cranial growth and the cranio-caudal folding of the embryo are responsible for the proximity of the dorsal aortas to the primitive heart tube. This convergence process, along with the looping of the two ventral aortas, allows the connection between the two structures, resulting in the first pharyngeal arch artery [57,58,59,60,61].

During the fourth week, the dorsal aortas fuse next to the fourth thoracic to fourth lumbar somitic segments and form a single arterial tube. In the cranial segment, they remain separated, attached to the aortic sac by aortic arches [55,57,58,59,60,61,62].

Between the fourth and fifth week, four more aortic arch pairs named 2 to 6 develop from the aortic sac. In fact, the fifth pair does not develop [55], or it completely regresses very early [57]. The development of the aortic arches is connected with the development of the pharyngeal arches, and, in addition, the arterial structures are found inside the mesenchyme of the pharyngeal arches [57]. The aortic arches, the same as pharyngeal arches, do not coexist. They are formed starting with the cranial segment towards the caudal one, and, while one is forming, the previously appeared one regresses [55,57,58,59,60,62].

The first aortic arch is the primordial connection between the aortic sac and dorsal aorta. It will regress starting from the twenty-eighth day, coexisting briefly with the second arch. Only a few parts persist as segments of the maxillary artery [60,62].

The second aortic arch forms on the twenty-sixth day and regresses on day twenty-nine. Its remnants form segments of the stapedius artery [59,62].

The appearance of the third, fourth, and sixth aortic arches occurs approximately in the same period. The third and fourth pairs appear on day twenty-eight, while the sixth pair appears on the twenty-ninth day. On the thirty-fifth day, the segment of the dorsal aorta between pairs three and four, called the carotid duct, disappears bilaterally. Thus, the third aortic arch will create the only connection between the aortic sac and the cranial segment of the two dorsal aortas. Consequently, it will supply blood to the cephalic extremity of the embryo [55]. Remnants of the third aortic arch form the common carotid arteries and the proximal segment of the internal carotid arteries. The cranial segment of the dorsal aorta persists bilaterally as the cranial segment of the internal carotid arteries. The external carotid arteries arise from the common carotid arteries following the angiogenesis process [55,57].

After the regression of the carotid duct, the fourth aortic arch remains connected with the distal segment of the corresponding dorsal aorta. The two seventh cervical intersegmental arteries (SCIA) are formed at the junction of the two dorsal aortas. The heart migration associated with the elongated embryo is responsible for the rising of the origin of these branches to maintain the correspondence with the upper limb somites. By the seventh week, the segment of the RDA between the origin of the right SCIA and the place of fusion with the LDA degenerates. The remaining segment of the right dorsal aorta connects the right fourth aortic arch to the right SCIA to form the right subclavian artery. The initial segment of the fourth aortic arch and the adjacent aortic sac structures will form the brachiocephalic artery [55,58,59,62]. On the left side, the fourth aortic arch remains connected to the aortic sac. It gives rise to the ascending aorta, and the cross and the proximal segment of the descending aorta. The distal segment of the descending aorta is formed by a fusion between the LDA and RDA. The left SCIA builds the left subclavian artery to supply the left upper limb [55,59,62].

The intersegmental arteries are born through the process of vasculogenesis. They appear in the somitic mesenchyme and further connect to the ipsilateral dorsal aorta. Like the somites, they have segmental organization: cervical, dorsal, lumbar, and sacral. The cervical intersegmental arteries form a complex anastomose centered by a longitudinal branch. In order to become the vertebral artery, it loses connection with the ipsilateral dorsal aorta, maintaining the connection with the seventh cervical intersegmental artery. This way, the origin of the vertebral artery corresponds to the subclavian artery [55].

The sixth aortic arch appears from the proximal part of the aortic sac and connects to the paired dorsal aorta caudally by the fourth aortic arch. It is the last to develop but the first to remodel—the septation and rotation of the outflow tract cause the late process. The development of the sixth aortic arch is not symmetric. On the right side, the aortic arch elongates due to the rotation process and loses connection with RDA while it connects with the artery arising from the pulmonary primordium [4,57,61,62]. On the left side, the connection between the sixth aortic arch and LDA persists during intrauterine life as the ductus arteriosus or Botalli’s duct. Eventually, it obstructs after birth, and its remains form the arterial ligament. Ventrally, it sprouts and extends towards the pulmonary bud. In order to form the pulmonary artery, it anastomoses with the vessels developed from the mesenchyme around to the bronchi [57,61]. Schoenwolf GC et al. [55] suggest that the main origin of the pulmonary artery is, in fact, the fourth aortic arch. During development, it loses its connection with the fourth aortic arch, but not before forming a connection with the sixth aortic arch.

### 4.2. The Complex Developmental Defects—Starting Point for an Original Morphological Classification

From the perspective of its course, ARSA can be situated retroesophageally (80–84%), between the esophagus and trachea (12.7–15%), or pretracheally (4.2–5%) [63,64,65]. At the same time, variants in the origin of the right common carotid artery and, respectively, of the right vertebral artery frequently accompanied ARSA. The bicarotid trunk, also known as truncus bicaroticus, has an incidence of 5%, the higher rates (4–20.6%) recorded in association with ARSA [65,66]. RVA often emerges from the right aberrant subclavian or common carotid artery. Only a few cases in which RVA originates in the left vertebral artery or directly in the aortic arch are reported [65]. Considering these facts, we propose an original classification that includes various morphological aspects, with the essential point represented by the ARSA trajectory. Compared to Adachi and Williams [67], our classification is more morphologically and clinically oriented:

Type I—retroesophageal course;
Type II—intertracheo-esophageal course;
Type III—pretracheal course;
a—origin of right vertebral artery from the aberrant right subclavian artery;
b—origin of right vertebral artery from the right common carotid artery;
c—origin of right vertebral artery from the aortic arch;
d—origin of right vertebral artery from the left vertebral artery;
B—presence of bicarotid trunk;
K—presence of Kommerell diverticulum.

### 4.3. Summary of Evidence

This systematic review included 47 case reports and case series with a total of 51 patients diagnosed with ARSA. Our main goal was to identify the morphological aspects of the lusoria artery, as well as the associated malformations. In accordance with the morphological types encountered, we propose a new classification system; the cases selected in the screening stage were entered in this classification. Secondarily, we analyzed the demographic distribution of the selected patients.

After analyzing the recorded data (Table 1), we identified a higher incidence of ARSA in women at 62.74% (32 out of 51) compared to men at 37.25% (19 out of 51). These data are in accordance with those presented by Polguj et al. [68], but the percentage difference recorded between the two sexes is smaller (55.3% vs. 44.7%).

The demographic analysis showed a relatively uniform distribution with a peak in the “44 to 57 years” and “58 to 71 years” age groups. These data are consistent with the distribution of specific symptoms with a high weight in the same age ranges. The non-specific symptomatology predominates in the “58 to 71 years” interval with minimum values in the “16 to 29 years” and “30 to 43 years” intervals.

Our research proves that the typical course of ARSA is retroesophageal (49 out of 51). Only two cases reported by Chen et al. [14] presented a pretracheal trajectory. Natsis et al. [64] mentions in his dissection study the presence of the intertracheo-esophageal path in 1 out of 6 cadavers without identifying the pretracheal path. ARSA is relatively frequently associated with Kommerell’s diverticulum, which can be found in 20–60% of the subjects who present this malformation, while the *truncus bicaroticus* is rarely found [69]. In our review, 15 out of 51 cases report the presence of Kommerell’s diverticulum, while the bicarotid trunk we identified only in seven cases. In a single case reported by Downey et al. [47], the association between the bicarotid trunk and the Kommerell diverticulum was identified. In the vast majority of cases, we identified the origin of the right vertebral artery to be in the ARSA (44 out of 51). In two cases, we found its origin in the aortic arch and five other cases in the ipsilateral common carotid artery. In the selected time interval, no case was reported in which the origin of the right vertebral artery was in the left vertebral artery. We present the cases frequencies included in our new classification in tabular form (Table 6).

Computed tomography angiography with 3D reconstruction techniques represents the gold standard for diagnosing ARSA, having a sensitivity of 100%. The transthoracic echocardiography detects ARSA in 92 out of 100 patients, while chest radiography identifies this malformation in only 20% of cases [68]. New techniques such as Photon-Counting CT ensure good image quality and an excellent contrast-to-noise ratio. In this context, patients with renal failure can benefit from the investigation, reducing the risks to a minimum, by reducing the amount of contrast material administered [70].

The occurrence of clinical manifestations varies between 7–10% [68] and 30% [44]. They are various but, at the same time, non-specific, with dysphagia (hence the name dysphagia lusoria), dyspnea, retrosternal pain, chest pain, or weight loss predominating. Our review is in accordance with the clinical aspects mentioned; the symptomatology is dominated by dysphagia (24 out of 51 subjects). Only 4 out of 51 patients report weight loss, being overtaken by chest pain (5 out of 51) and asymptomatic patients (7 out of 51). In addition, symptoms are the prerogative of adulthood, with symptoms appearing at approximately 48 years, with significant differences between men (44.9 years) and women (54 years). Several authors such as Osiro et al. [71] and Mochizuki et al. [72] associate ARSA with hemodynamic changes at the level of the upper limb, as well as at the vertebro-basilar level. The “subclavian steal syndrome” is clinically characterized by both central and peripheral ischemic and neurological phenomena.

Moreover, the morphological aspect of ARSA, such as the trajectory and coexisting vascular malformations (*truncus bicaroticus*), also determines the age of the onset of symptoms [69]. The predominance of wheezing, stridor, and recurrent lung infections are the prerogative of small children and are primarily due to the histological aspect of the trachea, which is easily compressible [73]. According to Polguj et al. [68], atherosclerosis is the main factor correlated with the appearance of symptoms in adults. In support of this statement, we point out that there is a concordance between the age of onset of ARSA symptoms and the age of onset of symptoms caused by atherosclerosis. This agreement is achieved both in the case of women, who are protected by the secretion of estrogen hormones, and in men.

The treatment must be staged and adapted to the morphological and clinical aspects. Follow-up is recommended for asymptomatic patients. Patients with minor and moderate clinical manifestations require lifestyle changes in terms of physical effort and eating habits. Surgical intervention should be considered in cases that show a significant decrease in the quality of life. Success depends on the malformation’s morphological aspect, the associated pathology, and the skills of the operative team [74,75].

Our study has some limitations. First, the small number of cases selected by the chosen protocol does not fully cover the proposed classification. This is based on the embryological evidence and the other articles studied during the elaboration of the manuscript. The classification system will be the basis of future research. Second, the chosen protocol only includes case reports and case series. We chose these inclusion and exclusion criteria in order to be able to benefit from cases where we can identify with certainty the morphological characters followed.

## 5. Conclusions

The aberrant right subclavian artery is a congenital anomaly determined by a developmental defect at the right fourth aortic arch level. The classification proposed by us achieves a complete framing of the morphological types of ARSA, as well as its association with the Kommerell diverticulum, the bicarotid trunk, and the aberrant origin of the right vertebral artery. This great variability must be considered by clinicians in therapeutic management. The presence of symptomatology in a small number of patients and the fact that it is non-specific make many cases incidental findings. In this sense, imaging studies such as computer tomography play a defined diagnostic role.

## Figures and Tables

**Table 1:** Inclusion and exclusion criteria.

Inclusion Criteria	Exclusion Criteria
1. Publications regarding aberrant right subclavian artery	1. Studies that do not contain a complete morphological description
2. Full text available	2. Only abstract available
3. Case reports and case series	3. Reviews studies
4. Manuscript in English	4. Retrospective studies
5. Human studies	

**Table 2:** Summary of studies presenting morphological aspects.

Author	Year	Gender	Age	ARSA Morphologic Aspect	Kommerell Diverticulum	Origin of RVA	Bicarotid Trunk	Simptoms
Akilu et al. [9]	2023	m	50	retroesophageal	present	ARSA	absent	shortness of breath, chest pain, dizziness
Nguyen et al. [10]	2023	f	55	retroesophageal	present	ARSA	absent	numbness, paresthesia, pain in the finger
Uemura et al. [11]	2023	f	54	retroesophageal	present	ARSA	absent	chest pain
Boukobza et al. [12]	2023	f	23	retroesophageal	absent	ARSA	present	ischemic stroke
Wawak et al. [13]	2023	f	24	retroesophageal	absent	AA	absent	dysphagia
Chen et al. [14]	2023	f	65	pretracheal	present	RCCA	absent	headaches
Chen et al. [14]	2023	m	60	pretracheal	present	RCCA	absent	headaches
Kavaliunaite et al. [15]	2023	m	50	retroesophageal	present	ARSA	absent	dysphagia
Ezemba et al. [16]	2023	m	28	retroesophageal	absent	ARSA	absent	dysphagia, foreign body sensation
Heye et al. [17]	2023	f	18	retroesophageal	present	ARSA	absent	dysphagia
Heye et al. [17]	2023	f	13	retroesophageal	absent	ARSA	absent	dysphagia
Uchino et al. [18]	2023	f	34	retroesophageal	absent	ARSA	present	absent
Lei et al. [19]	2023	f	69	retroesophageal	absent	ARSA	present	dizziness, blurred vision
Nasser et al. [20]	2023	f	82	retroesophageal	present	AA	absent	absent
Ostrowski et al. [21]	2023	m	63	retroesophageal	absent	RCCA	absent	absent
Mawait et al. [22]	2023	f	50	retroesophageal	present	ARSA	absent	dyspnea, dysphagia
Chan et al. [23]	2023	f	41	retroesophageal	absent	ARSA	absent	retrosternal pain, dysphagia
Iijima et al. [24]	2023	f	58	retroesophageal	absent	ARSA	present	cough, yellow sputum
Michos et al. [25]	2023	f	62	retroesophageal	absent	ARSA	absent	dysphagia
Misevicine et al. [26]	2023	f	1	retroesophageal	present	ARSA	absent	wheezing, cough
Tanaka et al. [27]	2023	m	84	retroesophageal	absent	ARSA	absent	absent
Najafi et al. [28]	2023	f	22	retroesophageal	absent	ARSA	absent	dysphagia
Solano et al. [29]	2023	m	54	retroesophageal	present	ARSA	absent	dyspnea, dysphagia
Bolaji et al. [30]	2023	f	58	retroesophageal	absent	ARSA	absent	retrosternal pain, dysphagia
Dugas et al. [31]	2023	f	1	retroesophageal	absent	ARSA	absent	emesis, poor weight gain
Erhiawarie et al. [32]	2023	f	66	retroesophageal	present	ARSA	absent	throat pain, dysphagia, weight loss
An et al. [33]	2023	f	13	retroesophageal	absent	ARSA	absent	dysphagia
Marey et al. [34]	2023	f	20	retroesophageal	present	ARSA	absent	dysphagia
Subramanian et al. [35]	2022	f	14	retroesophageal	absent	ARSA	absent	esophageal fistula
Chen et al. [36]	2022	f	34	retroesophageal	absent	ARSA	absent	hoarseness, dysphagia
Ada at al. [37]	2022	m	42	retroesophageal	absent	ARSA	present	dysphagia
Cook et al. [38]	2022	f	82	retroesophageal	present	ARSA	absent	upper abdominal pain
Cook et al. [38]	2022	m	61	retroesophageal	present	ARSA	absent	chest pain
Giglio et al. [39]	2022	f	72	retroesophageal	absent	ARSA	absent	paresthesia
Nakamura et al. [40]	2022	m	66	retroesophageal	absent	ARSA	absent	chest pain
Nakamura et al. [41]	2022	m	60	retroesophageal	absent	ARSA	absent	dysphagia, weight loss
Uganabo et al. [42]	2022	f	56	retroesophageal	absent	ARSA	absent	dysphagia, chest pain, peptic ulcer
Uganabo et al. [42]	2022	f	60	retroesophageal	absent	ARSA	absent	dysphagia
Dulam et al. [43]	2022	f	69	retroesophageal	absent	ARSA	absent	dysphagia, dyspepsia, esophageal reflux
Mansour et al. [44]	2022	f	54	retroesophageal	absent	ARSA	absent	dysphagia, chest pain, weight loss
El-Helou et al. [45]	2022	m	40	retroesophageal	absent	ARSA	absent	absent
Patil et al. [46]	2022	m	45	retroesophageal	absent	RCCA	absent	blackening of the right third finger
Downey et al. [47]	2022	m	47	retroesophageal	present	ARSA	present	uncontrolled hypertension, back pain
Clemente et al. [48]	2022	m	2	retroesophageal	absent	ARSA	absent	scimitar syndrome
Venkastesan et al. [49]	2022	m	57	retroesophageal	absent	ARSA	absent	absent
Kuwazoe et al. [50]	2022	f	76	retroesophageal	absent	ARSA	absent	dysphagia
Marsafi et al. [51]	2022	f	55	retroesophageal	absent	ARSA	present	dysphagia
Hlayhel et al. [52]	2022	m	71	retroesophageal	absent	ARSA	absent	severe respiratory distress
Murukendiran et al. [53]	2022	f	41	retroesophageal	absent	ARSA	absent	dysphagia
Venkatesan et al. [49]	2022	m	57	retroesophageal	absent	ARSA	absent	weakness
Spath et al. [54]	2022	m	66	retroesophageal	present	ARSA	absent	dysphagia

Abbreviations: f—female; m—male; RVA—right vertebral artery; AA—aortic arch; RCCA—right common carotid artery; ARSA—aberrant right subclavian artery.

**Table 3:** Distribution of frequencies by age groups.

No.	Age Groups	Frequencies	Relative Frequencies	Cumulative Absolute Frequencies	Cumulative Relative Frequencies
1	1–15 years	6	0.12	6	0.12
2	16–29 years	6	0.12	12	0.24
3	30–43 years	6	0.12	18	0.35
4	44–57 years	13	0.25	31	0.61
5	58–71 years	15	0.29	46	0.90
6	72–84 years	5	0.10	51	1.00

**Table 4:** Frequency distribution of specific symptoms by age group.

No.	Age Groups	Frequencies	Relative Frequencies	Cumulative Absolute Frequencies	Cumulative Relative Frequencies
1	1–15 years	2	0.08	2	0.08
2	16–29 years	4	0.15	6	0.23
3	30–43 years	4	0.15	10	0.38
4	44–57 years	8	0.31	18	0.69
5	58–71 years	7	0.27	25	0.96
6	72–84 years	1	0.04	26	1.00

**Table 5:** Frequency distribution of non-specific symptoms by age group.

No.	Age Groups	Frequencies	Relative Frequencies	Cumulative Absolute Frequencies	Cumulative Relative Frequencies
1	1–15 years	4	0.16	4	0.16
2	16–29 years	2	0.08	6	0.24
3	30–43 years	2	0.08	8	0.32
4	44–57 years	5	0.20	13	0.52
5	58–71 years	8	0.32	21	0.84
6	72–84 years	4	0.16	25	1.00

**Table 6:** Frequency distribution of morphologic types.

	Non-B Non-K				B				K				B + K			
TYPE	a	b	c	d	a	b	c	d	a	b	c	d	a	b	c	d
I	26	1	1	0	1	1	1	0	13	0	0	0	1	0	0	0
II	0	0	0	0	0	0	0	0	0	0	0	0	0	0	0	0
III	0	2	0	0	0	0	0	0	0	0	0	0	0	0	0	0

Abbreviations: B—truncus bicaroticus; K—Kommerall diverticulum.

## Data Availability

All relevant data are contained within the manuscript.

## References

[1] Brauner E., Lapidot M., Kremer R., Best L.A., Kluger Y. (2011). Aberrant right subclavian artery—Suggested mechanism for esophageal foreign body impaction: Case report. World J. Emerg. Surg..

[2] Asherson N. (1979). David Bayford. His syndrome and signs of dysphagia lusoria. Ann. R. Coll. Surg. Engl..

[3] Carrizo G.J., Marjani M.A. (2004). Dysphagia lusoria caused by an aberrant right subclavian artery. Tex. Heart Inst. J..

[4] Agati S., Sousa C.G., Calvaruso F.D., Zanai R., Campanella I., Poli D., Di Pino A., Borro L., Iorio F.S., Raponi M. (2019). Anomalous aortic origin of the pulmonary arteries: Case series and literature review. Ann. Pediatr. Cardiol..

[5] Tarniceriu C.C., Hurjui L.L., Tanase D.M., Nedelcu A.H., Gradinaru I., Ursaru M., Stefan Rudeanu A., Delianu C., Lozneanu L. (2021). The Pulmonary Venous Return from Normal to Pathological-Clinical Correlations and Review of Literature. Medicina.

[6] Coles M., Madray V.M., Mareddy C., Kapoor D., Sharma A. (2020). Dysphagia lusoria: A vascular etiology?. JGH Open.

[7] Cai M., Lin N., Fan X., Chen X., Xu S., Fu X., Xu L., Huang H. (2022). Fetal Aberrant Right Subclavian Artery: Associated Anomalies, Genetic Etiology, and Postnatal Outcomes in a Retrospective Cohort Study. Front. Pediatr..

[8] Page M.J., McKenzie J.E., Bossuyt P.M., Boutron I., Hoffmann T.C., Mulrow C.D., Shamseer L., Tetzlaff J.M., Akl E.A., Brennan S.E. (2021). The PRISMA 2020 statement: An updated guideline for reporting systematic reviews. BMJ.

[9] Akilu W., Feng Y., Zhang X.X., Li S.L., Ma X.T., Hu M., Cheng C. (2023). Carotid-subclavian bypass and endovascular aortic repair of Kommerell’s diverticulum with aberrant left subclavian artery: A case report. World J. Clin. Cases.

[10] Nguyen T.N., Girard M., Cintron D., Perez V., Patton M. (2023). A bird in the hand: Acute digital ischemia due to aberrant subclavian artery. J. Am. Coll. Cardiol..

[11] Uemura H., Matsue H., Suehiro Y., Nakagawa T., Satoh A., Satoh H. (2023). Open surgical repair of Stanford type A dissection due to Kommerell’s diverticulum associated with an aberrant right subclavian artery. Ann. Vasc. Surg.—Brief Rep. Innov..

[12] Boukobza M., Laissy J.-P. (2023). In-vivo imaging of a rare constellation of arterial variants: Aberrant subclavian artery, bicarotid trunk, and ectopic vertebral arteries. Neuroradiol. J..

[13] Wawak M., Pieniążek P., Tekieli Ł., Paluszek P., Trystuła M., Przewłocki T., Kabłak-Ziembicka A. (2024). Coarctation of the aorta, carotid artery stenosis and aberrant right subclavian artery as a rare cause of cerebral ischemia in a primigravid woman. Quant. Imaging Med. Surg..

[14] Chen J., Liu L., Kou X., Wang C. (2023). Case report: Right vertebral and carotid artery anomalies with an aberrant right subclavian artery in two patients. Front. Neurol..

[15] Kavaliunaite E., Kjeldsen B.J., Midtgaard A., Akgul C. (2023). Complex Hybrid Repair of an Aberrant Right Subclavian Artery With Rapidly Developing Kommerell’s Diverticulum. EJVES Vasc. Forum.

[16] Ezemba N., Onuh A.C., Onoh U.S. (2023). Incomplete vascular ring of the aortic arch presenting with dysphagia in an adult: Case report. Pan Afr. Med. J..

[17] Heye T., Greiten L., Story-Hefta L., Reemtsen B., Moursi M. (2023). Aberrant right subclavian artery: A novel approach and an overview of operative techniques. J. Vasc. Surg. Cases Innov. Tech..

[18] Uchino A., Kondo R. (2023). Medial-type persistent trigeminal artery and ipsilateral posterior communicating artery supplying bilateral posterior cerebral arteries combined with an aberrant right subclavian artery and bicarotid trunk. Surg. Radiol. Anat. SRA.

[19] Lei M.X., Cao Y., Yuan M.Q. (2024). Aberrant right subclavian artery associated with common carotid trunk. J. Vasc. Surg..

[20] Nasser M., Petrocheli B.B., Felippe T.K.S., Isola B., Dos Santos Pereira B.C., Sartoreli A.L.C., Batista J.M., Brandão G.M.S. (2023). Aberrant right subclavian artery: Case report and literature review. J. Vasc. Bras..

[21] Ostrowski P., Bonczar M., Przybycień W., Zamojska I., Kołodziejczyk B., Walocha J., Koziej M. (2023). An aberrant right subclavian artery in a 63-year-old male cadaver. Folia Morphol..

[22] Mawait N., Kerzmann A., Desiron Q., Henroteaux D., Stiennon L., Defraigne J.O. (2023). «Dysphagia lusoria» traitée chirurgicalement [Dysphagia lusoria treated by surgery]. Rev. Med. Liege.

[23] Chan S., Fermanis G. (2023). Translocation of aberrant right subclavian artery to the ascending aorta-a treatment for dysphagia lusoria. J. Surg. Case Rep..

[24] Iijima Y., Ishikawa M., Iwai S., Yamagata A., Motono N., Tsuji H., Uramoto H. (2022). Usefulness of intraoperative nerve monitoring for giant type AB thymoma combined with an aberrant right subclavian artery: A case report. J. Cardiothorac. Surg..

[25] Michos L., Hart C., Nantermet S., Meisner R. (2023). Surgical repair of severe dysphagia lusoria. J. Vasc. Surg. Cases Innov. Tech..

[26] Miseviciene V., Liakaite G., Zaveckiene J., Snipaitiene A. (2023). Case report: Unilateral pulmonary artery agenesis and Kommerell’s diverticulum in 1-year old girl. Front. Pediatr..

[27] Tanaka T., Sashida R., Hirokawa Y., Matsuno A. (2023). Aberrant Right Subclavian Artery Identified before Carotid Artery Stenting. Jpn. Med. Assoc. J..

[28] Najafi M.J., Davoodi M., Godazandeh G.A., Zahmatkesh A., Faghani N., Bashiri R. (2023). Minimally invasive approach in symptomatic aberrant right subclavian artery treatment. Int. J. Surg. Case Rep..

[29] Solano A., Pizano A., Azam J., Gonzalez-Guardiola G., Siah M., Chamseddin K., Prakash V., Kirkwood M.L., Shih M. (2023). Kommerell’s Diverticulum in a Right-Sided Aortic Arch with an Aberrant Left Subclavian Artery Hybrid Repair. Vasc. Endovasc. Surg..

[30] Bolaji O., Ouedraogo F., Adabale O.K., Ugoala O.S. (2023). Navigating the Esophageal Enigma: A Vascular Odyssey of Dysphagia. Cureus.

[31] Dugas J., Vozar A., Deskins S.J., Udassi S. (2023). Infant With Recurrent Vomiting and Poor Weight Gain Secondary to an Aberrant Subclavian Artery. Cureus.

[32] Erhiawarie F., Chioma O.E., Rahim O., Ansar S., Adabanya U., Omole A.E., Awosika A. (2023). Right Subclavian Artery With Kommerell’s Diverticulum: A Rare Cause of Dysphagia. Cureus.

[33] An K.R., Deng M.X., Freud L.R., Honjo O. (2023). Repair of Aberrant Right Subclavian Artery Causing Dysphagia Lusoria via Partial Median Sternotomy. World J. Pediatr. Congenit. Heart Surg..

[34] Marey G., Fethke E., Said S.M. (2023). Single-incision, off-pump repair of a right aortic arch with an aberrant left subclavian artery and diverticulum of Kommerell in an adult. Multimed. Man. Cardiothorac. Surg..

[35] Subramanian P.C., Chidanandaswamy N., Soundararajan R., Bhujade H., Prabhakar N. (2022). An Aberrant Right Subclavian Artery-Esophageal Fistula-A Fatal Complication of a Common Anomaly: A Case Report and Review of Literature. Indian J. Radiol. Imaging.

[36] Chen Y.W., Chang S.L., Wu N.C., Shih Y.J. (2022). Ortner syndrome caused by aberrant right subclavian artery: A case report. Medicine.

[37] Ada F., Güler S., Demir F., Şahin E. (2022). A rare condition secondary to aberrant right subclavian artery syndrome: Aphagia lusoria. Turk. J. Thorac. Cardiovasc. Surg..

[38] Cook V., Singla A.A., Herlihy D., Chui J., Fisher C., Puttaswamy V. (2022). Involvement of Kommerell’s diverticulae-a new anatomical risk factor for acute aortic syndrome progression and technical considerations. J. Surg. Case Rep..

[39] Giglio V., Badar Z., Bhogadi Y., Van Adel B., Yip G. (2022). Aortosternal Venous Compression: A Review of Two Cases. Case Rep. Med..

[40] Nakamura Y., Kumada Y., Mori A., Kawai N., Ishida N., Kasugai T. (2022). Thoracic endovascular aortic repair for chronic aortic dissection after total arch replacement for aberrant right subclavian artery: A case report. SAGE Open Med. Case Rep..

[41] Nakamura Y., Imaoka S., Yamakura T., Yamasumi T., Kondoh H. (2022). Thoracic Endovascular Aortic Repair With Subclavian Revascularization for Symptomatic Nonaneurysmal Aberrant Right Subclavian Artery. Tex. Heart Inst. J..

[42] Ugonabo O., Mohamed M., Frandah W., Sherif A. (2022). Two Patients with Difficulty in Swallowing due to Dysphagia Lusoria. J. Med. Cases.

[43] Dulam V., Keshavamurthy S., Kumaran M., Hota P., Gutierrez C., Kashem M.A., Toyoda Y. (2022). Caught in a vice. Indian J. Thorac. Cardiovasc. Surg..

[44] Mansour M.M., Darweesh M., Mahfouz R., Obeidat A.E., Das K. (2022). Aberrant Right Subclavian Artery Causing Dysphagia: A Case Report of Dysphagia Lusoria. Cureus.

[45] El-Helou E., Zaiter M., Shall A., Sleiman Y., Liberale G., Pop C.F. (2022). Persistent Left Superior Vena Cava Associated with Right Aberrant Subclavian Artery Detected during Totally Implantable Vascular Access Device Insertion. Surg. J..

[46] Patil C., Kotamraju S., Kumar P., Kollu R., Reddy M. (2022). Right Vertebral Artery Arising From Ipsilateral Common Carotid Artery With Severe Stenosis of Ostio-proximal Segment of Aberrant Right Subclavian Artery: A Rare Life-Saving Variant. Cureus.

[47] Downey P.S., Thors A., Johnson P., Gupta K., Wallisch W.J., Almoghrabi O., Muehlebach G.F., Zorn G.L. (2022). Hybrid repair of acute type B dissection with aberrant right subclavian artery and bicarotid trunk. J. Vasc. Surg. Cases Innov. Tech..

[48] Clemente A., Viganò G., Festa L., Remoli E., Marrone C., Federici D., Pak V., Chiappino D., Santoro G., Ait-Ali L. (2022). Multimodality Approach to a Complex Scimitar Syndrome: How Advanced Diagnostics Can Guide Therapeutic Strategies. JACC Case Rep..

[49] Venkatesan A., Gonuguntla A., Vasireddy A., Rai G.D., Kamath G.S., Bishnoi A.K., Maramreddy R. (2022). Asymptomatic Giant Aneurysm of the Arteria Lusoria Treated by Debranching and Aneurysmal Resection. Vasc. Spec. Int..

[50] Kuwazoe H., Enomoto K., Murakami D., Kumashiro N., Takeda S., Hotomi M. (2022). The Role of Anatomical Imaging and Intraoperative Neuromonitoring (IONM) for Successful Prediction of a Nonrecurrent Laryngeal Nerve. Case Rep. Surg..

[51] Marsafi O., Chahbi Z., Wakrim S. (2021). When Arteria lusoria meets Truncus bicaroticus: One of the rarest combinations of aortic arch anomalies. Radiol. Case Rep..

[52] Hlayhel A., Foran L., Trivedi A., Zuberi J., Cerda L., Danks J. (2022). A case of esophageal ulcer and hemorrhage due to aberrant subclavian in a COVID positive patient. J. Surg. Case Rep..

[53] Murukendiran G.J., Dash P.K., Azeez A.M., Palanisamy N., Pitchai S. (2022). Symptomatic aberrant right subclavian artery-A case report and anesthetic implications. Ann. Card. Anaesth..

[54] Spath P., Marazzi G., Stana J., Peterss S., Fernandez-Prendes C., Rantner B., Pichlmaier M.A., Tsilimparis N. (2022). Endovascular Repair With Triple Inner-Branch Endograft for Aberrant Subclavian Artery Aneurysm: A Case Report. J. Endovasc. Ther..

[55] Schoenwolf G.C., Bleyl S.B., Brauer P.R., Francis-West P.H. (2014). Development of the Vasculature. Larsen’s Human Embryology.

[56] Nedelcu A.H., Ţepordei R.T., Sava A., Stan C.I., Aignătoaei A.M., Ţăranu T., Ursaru M. (2016). Supernumerary fronto-orbital arteries arising from contralateral anterior cerebral artery associated with partially duplicated anterior communicating artery—Case study and literature review. Rom. J. Morphol. Embryol..

[57] Khalid N., Bordoni B. (2023). Embryology, Great Vessel. StatPearls.

[58] Kau T., Sinzig M., Gasser J., Lesnik G., Rabitsch E., Celedin S., Eicher W., Illiasch H., Hausegger K.A. (2007). Aortic development and anomalies. Semin. Interv. Radiol..

[59] Qiu Y., Wu X., Zhuang Z., Li X., Zhu L., Huang C., Zhuang H., Ma M., Ye F., Chen J. (2019). Anatomical variations of the aortic arch branches in a sample of Chinese cadavers: Embryological basis and literature review. Interact. Cardiovasc. Thorac. Surg..

[60] Hiruma T., Nakajima Y., Nakamura H. (2002). Development of pharyngeal arch arteries in early mouse embryo. J. Anat..

[61] Anderson R.H., Bamforth S.D. (2022). Morphogenesis of the Mammalian Aortic Arch Arteries. Front. Cell Dev. Biol..

[62] Kellenberger C.J. (2010). Aortic arch malformations. Pediatr. Radiol..

[63] Myers P.O., Fasel J.H., Kalangos A., Gailloud P. (2010). Arteria lusoria: Developmental anatomy, clinical, radiological and surgical aspects. Ann. Cardiol. Angeiol..

[64] Natsis K., Didagelos M., Gkiouliava A., Lazaridis N., Vyzas V., Piagkou M. (2017). The aberrant right subclavian artery: Cadaveric study and literature review. Surg. Radiol. Anat..

[65] Buffoli B., Verzeletti V., Hirtler L., Rezzani R., Rodella L.F. (2021). Retroesophageal right subclavian artery associated with a bicarotid trunk and an ectopic origin of vertebral arteries. Surg. Radiol. Anat..

[66] Natsis K., Piagkou M., Lazaridis N., Kalamatianos T., Chytas D., Manatakis D., Anastasopoulos N., Loukas M. (2021). A systematic classification of the left-sided aortic arch variants based on cadaveric studies’ prevalence. Surg. Radiol. Anat..

[67] Leite T.F.O., Pires L.A.S., Cisne R., Babinski M.A., Chagas C.A.A. (2017). Clinical discussion of the arteria lusoria: A case report. J. Vasc. Bras..

[68] Polguj M., Chrzanowski Ł., Kasprzak J.D., Stefańczyk L., Topol M., Majos A. (2014). The aberrant right subclavian artery (arteria lusoria): The morphological and clinical aspects of one of the most important variations--a systematic study of 141 reports. Sci. World J..

[69] Van Rosendael P.J., Stöger J.L., Kiès P., Vliegen H.W., Hazekamp M.G., Koolbergen D.R., Lamb H.J., Jongbloed M.R.M., Egorova A.D. (2021). The Clinical Spectrum of Kommerell’s Diverticulum in Adults with a Right-Sided Aortic Arch: A Case Series and Literature Overview. J. Cardiovasc. Dev. Dis..

[70] Zanon C., Cademartiri F., Toniolo A., Bini C., Clemente A., Colacchio E.C., Cabrelle G., Mastro F., Antonello M., Quaia E. (2024). Advantages of Photon-Counting Detector CT in Aortic Imaging. Tomography.

[71] Osiro S., Zurada A., Gielecki J., Shoja M.M., Tubbs R.S., Loukas M. (2012). A review of subclavian steal syndrome with clinical correlation. Med. Sci. Monit..

[72] Mochizuki Y., Mikawa S., Kutara K., Sugimoto K., Sakoya H., Ohnishi A., Saeki K., Shimizu Y., Kanda T., Asanuma T. (2021). Local Hemodynamic Changes Immediately after Correction of an Aberrant Right Subclavian Artery in a Dog: A Contrast Computed Tomographic Study. Vet. Sci..

[73] Irakleidis F., Kyriakides J., Baker D. (2021). Aberrant right subclavian artery—A rare congenital anatomical variation causing dysphagia lusoria. Vasa.

[74] Fanelli U., Iannarella R., Meoli A., Gismondi P., Cella S., Vincenzi F., Esposito S. (2020). An Unusual Dysphagia for Solids in a 17-Year-Old Girl Due To a Lusoria Artery: A Case Report and Review of the Literature. Int. J. Environ. Res. Public Health.

[75] Nelson J.S., Hurtado C.G., Wearden P.D. (2020). Surgery for Dysphagia Lusoria in Children. Ann. Thorac. Surg..

